# The Effects of Different Feed Substrates on Production Performance, Digestibility, and Chemical Composition of *Zophobas morio* Larvae

**DOI:** 10.3390/insects17101049

**Published:** 2026-10-12

**Authors:** Miroslava Polovinski Horvatović, Igor Jajić, Marko Vukadinović, Miloš Petrović, Dejan Beuković, Nikola Laćarac, Mirko Ivković

**Affiliations:** Faculty of Agriculture, University of Novi Sad, Trg Dositeja Obradovića 8, 21000 Novi Sad, Serbia; miroslava.polovinski@stocarstvo.edu.rs (M.P.H.); igor.jajic@stocarstvo.edu.rs (I.J.); marko.vukadinovic@stocarstvo.edu.rs (M.V.); milos.petrovic@polj.edu.rs (M.P.); dejan.beukovic@stocarstvo.edu.rs (D.B.); nikola.lacarac@polj.edu.rs (N.L.)

**Keywords:** *Zophobas morio*, production performance, digestibility, chemical composition

## Abstract

*Zophobas morio* is a potentially promising insect species for mass rearing; however, available data on its production performance and nutritional utilization remain limited. In this study, larvae were reared on nine different feed substrates, of which seven were retained for complete quantitative analysis. Production performance was evaluated at both tray and the individual larva weight to reflect conditions relevant to intensive production systems. Significant differences in production performance were observed among diets. Wheat bran resulted in the best performance, with a feed conversion ratio (FCR) of 2.55, whereas breadcrumbs resulted in the poorest performance (FCR 17.50). In two treatments (soybean grits and field pea), production performance could not be evaluated due to severe mortality and cannibalistic behavior or complete mortality, respectively, and these treatments were excluded from further analyses. Digestibility parameters could not be determined for the breadcrumbs treatment due to extremely low feed intake, which was insufficient to allow excreta (frass) collection required for the digestibility trial. Digestible energy ranged from 5.54 MJ/kg dry matter in canola meal to 10.40 MJ/kg dry matter in wheat grain. Diet composition had a pronounced effect on larval chemical composition. It is possible that carbohydrate-rich diets were associated with higher lipid accumulation (up to 60.44% in breadcrumbs), whereas protein-rich substrates were associated with increased larval protein content (up to 59.79% in sunflower meal), indicating clear dietary modulation of nutrient deposition. Overall, the results confirm that both production efficiency and larval composition in *Zophobas morio* are strongly dependent on diet formulation, highlighting the importance of optimized feeding strategies for its potential industrial production.

## Simple Summary

*Zophobas morio*, commonly known as the superworm, is a promising insect for large-scale production because its larvae can be used as a source of nutrients and protein. However, information on how different feeds affect its growth, feed use, and nutritional value is still limited. In this study, larvae were reared on several different feed ingredients, including feeds rich in protein and carbohydrates. Growth and production performance were evaluated both per tray and based on individual larval growth. The type of feed had a clear effect on larval performance. Wheat bran supported the best overall production performance, whereas breadcrumbs resulted in the poorest performance. Some feeds, including soybean grits and field pea, were excluded from the trial due to severe mortality and cannibalistic behaviour and could not be evaluated further. Feed composition also strongly affected the nutritional composition of the larvae. Larvae fed carbohydrate-rich feeds tended to contain more fat, while those fed protein-rich feeds tended to contain more protein. These findings show that feed choice is an important factor in superworm production and provide useful information for developing more efficient feeding strategies for large-scale rearing.

## 1. Introduction

The continuously growing global human population has led to a steadily increased demand for protein, both for direct human consumption and, indirectly, through the intensification of animal production systems worldwide [1]. Within these systems, protein represents the single most expensive and resource-intensive component of animal feed, placing considerable economic pressure on producers. This is largely due to the fact that the primary protein-rich feed ingredients, most notably soybean meal and fish meal, are characterized by high and often volatile market prices, as well as limited and geographically concentrated availability [2]. Soybean cultivation competes with land resources and raises environmental concerns related to deforestation and freshwater use, while fish meal production is constrained by the finite biomass of wild fish stocks and the growing pressure on marine ecosystems [3,4]. As a result, the livestock and aquaculture sectors face mounting challenges in securing adequate supplies of high-quality, digestible protein at economically viable costs, a situation that is expected to worsen in the coming decades as both human population and per capita protein consumption continue to rise [5]. In Europe, insects are increasingly being studied as a promising alternative protein source [6], while also gaining attention within the broader framework of the circular economy concept [7]. Beyond their nutritional value, insects offer notable environmental advantages—most significantly, their production is associated with substantially lower greenhouse gas emissions compared to conventional livestock farming [8]. This combination of high protein potential and a considerably reduced environmental footprint positions insects as a particularly attractive and sustainable component of future food and feed systems. European food safety authority (EFSA) has assessed that certain insect species are safe for both human consumption and as a feedstuff in animal husbandry under the specified conditions of use [9,10,11,12]. These species were selected based on prior experience in rearing and established production technologies, while it is expected that other insect species will, in the future, be authorized as novel foods following appropriate safety assessments. In addition, a significantly larger number of insect species are already permitted for use in animal feed than in human food, under feed regulatory frameworks. In Serbia, insect production is currently permitted only for use in animal feed [13].

Although *Zophobas morio* shows good production and nutritional potential, this species has not yet been included in the EU novel food assessment for various regulatory and research-related reasons, although this does not mean that it will not be considered in the future. Research on this insect species is developing in two directions: as a potential source of protein in animal nutrition [14,15,16,17,18] and as a model for the biodegradation and processing of plastic materials [18].

*Tenebrio molitor* and *Zophobas morio* are frequently studied together in the literature, as they belong to the same family (*Tenebrionidae*) and are often considered comparable model species for insect-based protein production [6,19]. In that context, it can be noted that although *Zophobas morio* may achieve higher individual larval masses, overall growth and production indicators suggest that it is currently less economically efficient under existing rearing conditions [20]. In this study, comparing insect species reared on a control diet and four experimental diets differing in protein and starch content, *Tenebrio molitor* generally showed higher feed conversion efficiency than *Zophobas morio* across most dietary treatments. Another limitation is that most experimental studies on insect rearing primarily focus on individual growth performance under controlled low-density conditions, often using a limited number of individuals per replicate. However, in industrial production systems, insects are reared at significantly higher densities, where social interactions, microclimatic conditions, and pathogen dynamics can strongly influence survival and productivity. Therefore, individual-level performance metrics may not fully reflect production-scale outcomes and economic feasibility. One of the other approaches is the study by Pascual [21], which reports both individual growth and total biomass gain per replicate, allowing for a more integrated assessment of production performance; however, such studies are still predominantly conducted on *Tenebrio molitor*.

The production performance of *Zophobas morio* is somewhat lower compared to some other industrially relevant insect species; however, these differences are not substantial enough to exclude its production potential. Considering that this species is less extensively studied and that available knowledge on its nutritional physiology is more limited, further optimization of feeding strategies may lead to additional improvements in its production performance.

As with other insect species, production parameters are largely dependent on the composition and nutritional quality of the diet [17,22,23,24]. Therefore, future research is expected to focus on a more detailed understanding of the species’ responses to individual feed components, as well as on optimizing the protein-to-energy ratio in the diet in order to improve the efficiency of feed conversion into biomass.

This study was conducted on *Zophobas morio* larvae, using nine different feed substrates. Therefore, this study aimed to evaluate the production performance of *Zophobas morio* at both tray and assessed individual weighs and to determine the apparent digestibility and digestible energy of several individual feedstuffs. Production performance, efficiency of conversion of ingested feed (ECI), total weight gain per container, individual weight gain, and the number of individuals were evaluated. Measurements were performed weekly, and the results were expressed on a weekly basis. In addition, the specific growth rate was calculated and expressed as a percentage. For two feed substrates, the experiment could not be completed: complete larval mortality was observed in the field pea treatment, while in the soybean grits treatment, only a few larvae remained due to pronounced cannibalism.

## 2. Materials and Methods

### 2.1. Rearing of Insects and Experimental Procedure

Insects used in this experiment were maintained at the Laboratory for Entomology, Department of Environmental and Plant Protection, Faculty of Agriculture. The rearing cycle of *Zophobas morio* was initiated using adults aged 10 days, which were introduced into a substrate prepared by combining wheat bran (80%), ground oat flakes (15%), and brewer’s yeast (5%). The substrate mixture was distributed evenly across plastic trays measuring 20 × 40 × 60 cm, into which egg cardboard boxes were placed to serve as shelter and to minimize aggressive interactions among individuals. Adults were allowed a 3-day period for copulation and oviposition, after which they were removed from the trays. The trays, together with the cardboard inserts, were subsequently transferred to a climate chamber maintained at 28 °C and 60% relative humidity for incubation. Throughout this initial four-week rearing period, larvae were supplemented with fresh slices of apple and carrot.

Upon reaching four weeks of age and a mean body weight of 0.030 g, larvae were sieved from the rearing substrate and transferred onto nine experimental diets, with approximately 67 larvae per tray. Each of the nine treatments consisted of four replicates, with each replicate containing 2 g of larvae (approximately 68 individuals) placed in a plastic tray measuring 11 × 6.5 × 5.5 cm (surface area: 60.5 cm^2^). All larvae were maintained under identical environmental conditions of 28 ± 1 °C and 65 ± 5% relative humidity, mostly in total darkness, except during sieving, feeding, or agar supplementation. Feedstuff was replenished based on visual assessment of consumption every other day, with additional feed provided when the previously supplied amount had been almost completely consumed, while the same amount of agar was added to each replicate every other day to provide moisture Agar was used as a moisture source instead of apple or carrot because, unlike these fresh plant materials, it provides water while introducing only a negligible amount of additional nutrients into the experimental system, thereby minimizing potential effects on larval nutrient intake and the evaluation of the experimental substrates.

From the point of diet assignment, larvae continued their development for an additional eight weeks, or until growth cessation was confirmed by two consecutive measurements showing no significant change in body weight. Larvae were counted and weighed as a group at weekly intervals, and the average individual larval weight was calculated by dividing the total larval weight by the number of larvae. The experimental endpoint was defined based on the completion of larval growth in the control treatment (wheat bran), which showed the fastest development among all diets. This approach was selected to reflect a production-oriented perspective, where rearing duration is a critical economic factor. Consequently, all other treatments were terminated simultaneously at this time point to enable comparison of production performance under equal rearing time conditions relevant to potential intensive production systems. At the end of the experimental period, after two days of fasting, larvae were sampled for chemical composition analysis. Each tray was treated as one independent biological replicate, and larvae collected from each tray were analyzed separately.

### 2.2. Feed

All feed ingredients were purchased locally, with soybean grits and field peas being commercially intended for animal feed; the soybean grits had undergone standard industrial processing prior to use. Before use, all feed ingredients were milled and then sieved through a 2 mm mesh to ensure particle size uniformity and eliminate clumping. Feed consumption was recorded weekly by monitoring the amount of feed provided and the residual feed in each tray. Weekly production performance parameters were calculated using the recorded number and total biomass of live larvae in each tray, together with the corresponding weekly feed consumption. Initially, relatively small amounts of feed (5 g per tray) were provided, and feed consumption was checked every 2–3 days. Based on the observed consumption, additional feed was provided as required, with the aim of avoiding excessive accumulation of uneaten feed. The amount of feed added was weighed using a precision balance (KERN EWJ 600-2M, KERN & SOHN GmbH, Balingen, Germany; readability 0.01 g; minimum weight 0.5 g). Any remaining feed was manually collected and weighed as accurately as possible. Feed consumption was calculated from the amount of feed provided and the amount of residual feed. However, two treatments—field pea and soybean grits—were excluded from further analysis due to severe survival and behavioral issues observed during the course of the trial. The remaining seven treatments were: wheat bran, canola meal, sunflower meal, breadcrumbs, wheat and triticale (milled whole grain) and dried distillers grains with solubles (DDGS). All calculations were performed on an as-fed basis (naturally dry matter). The following calculations were used to determine biomass gain, feed conversion and growth performance parameters:

Biomass gain was calculated as:

Biomass gain (g) = final live larval biomass − initial live larval biomass.

Feed conversion ratio (FCR, based on fresh weight) was calculated as:

FCR = feed disappearance (g)/biomass gain (g).

Efficiency of conversion of ingested food (ECI, based on fresh weight) was calculated as:

ECI (%) = [biomass gain (g)/feed ingested (g)] × 100.

Specific growth rate (SGR, based on fresh weight) was calculated as:

SGR (% day^−1^) = 100 × [ln(final body weight) − ln(initial body weight)]/experimental period (days).

FCR and ECI were calculated from feed intake and live biomass gain at the tray level. No correction for the biomass of dead or cannibalized larvae was applied, as such biomass losses could not be quantified reliably under group-rearing conditions.

The chemical composition of the feedstuffs, as determined by chemical analysis, is presented in Table 1.

### 2.3. Digestibility Trial

Apparent dry matter digestibility and digestible energy (DE) of the tested substrates were estimated using a group-based mass balance approach adapted from methodologies developed for *Tenebrio molitor* [21], with modifications to account for species-specific feeding behavior of *Zophobas morio* larvae. For the digestibility trial, 20 g of larvae of similar age and body weight were allocated to each replicate, with four replicates per feed substrate. Based on a preceding production trial, the amount of feed offered was adjusted according to the observed feed consumption of the larvae. Importantly, feed consumption differed among the tested substrates; therefore, the amount of feed supplied was adjusted accordingly to avoid both excessive feed availability and insufficient feed supply, while minimizing the risk of cannibalistic behavior. Feed intake was calculated as the amount of feed offered minus the amount of residual unconsumed feed recovered from each replicate. Frass was collected once weekly over three consecutive weeks and pooled for each replicate prior to digestibility assessment.

Larvae were reared in groups and provided with feed. Feed supply in the digestibility trial was adjusted based on prior intake data from the production experiment, and was offered in controlled portions to ensure near-complete consumption and accurate estimation of digestibility parameters. Frass was collected at the end of each interval, separated manually, and all samples were analyzed on a dry matter basis. The frass exhibited a consistent granular (pellet-like) structure, allowing clear visual distinction from unconsumed feed and facilitating careful separation. Nevertheless, complete physical separation of frass from small feed particles could not be guaranteed.

All calculations were performed at the group level. Apparent digestibility was estimated from the relationship between feed intake and frass output. During the experiment, selective feeding behavior was observed for certain substrates, including preferential consumption of inner fractions (e.g., sunflower meal) and delayed acceptance in the case of canola meal. Since all treatments were conducted under identical conditions, the method enables comparative evaluation of digestibility and digestible energy among substrates.

The apparent total tract digestibility coefficient (ATTD) of dry matter (DM) or energy was as follows:

ATTD = ((DM intake − DM frass)/(DM intake))

Digestible energy (DE) was determined as follows:

DE = (gross energy intake − gross energy of frass)/(feed intake (kg DM)

### 2.4. Chemical Analyses

Dry matter content was determined after drying [24]. Crude protein was analyzed according to the standard Kjeldahl method [25] conversion factor of 6.25, while crude fat content (ether extract) was determined as petroleum ether extract [26]. Ash content was determined in the furnace at 600 °C [27]. Nitrogen-free extract (NFE) was calculated by difference as dry matter minus crude protein, ether extract, ash, and crude fibre contents, with all components expressed on a natural dry matter basis. NDF (neutral detergent fiber) and ADF (acid detergent fiber) were determined on the ANKOM DELTA fiber analyzer (ANKOM Technology, Macedon, NY, USA) by applying methods provided by the manufacturer [28]. Hemicelluloses were calculated by subtracting ADF from NDF. Acid detergent lignin (ADL) was determined by treating the acid detergent fiber (ADF) residue with 72% sulfuric acid according to the method of Van Soest. Gross energy of the feed and frass samples was determined using an IKA oxygen bomb calorimeter (IKA^®^ Werke, Staufen, Germany) located at the Laboratory, Faculty of Agriculture, University of Novi Sad, Serbia. Digestible energy was subsequently calculated from the measured gross energy values of the feed and frass using the energy balance equation described below.

### 2.5. Statistical Analysis and Data Presentation Description

Statistical analysis was performed using one-way analysis of variance (ANOVA), followed by Tukey’s HSD test. Normality was assessed by visual inspection of normal probability plots of the model residuals. When the assumption of homogeneity of variances was violated, as indicated by a significant Levene’s test, Welch’s ANOVA was used, followed by Spjøtvoll–Stoline post hoc test. Statistical analyses were performed using Statistica 14.1.0.8 (TIBCO Software Inc., San Ramon, CA, USA), with the exception of mortality analysis. Mortality analysis was based on the numbers of dead and surviving larvae recorded for each tray, with each tray treated as an independent experimental unit. Mortality was analyzed using a generalized linear model assuming a quasibinomial distribution with a logit link. Pairwise comparisons among treatments were performed using estimated marginal means with Holm adjustment for multiple comparisons. This analysis was performed in R (version 4.6.1) using the emmeans package. Whole-trial results are presented in tabular form and constitute the basis for statistical inference, whereas weekly results are presented graphically as descriptive data.

## 3. Results

In the field pea treatment, a substantial proportion of larvae exhibited mortality during the early experimental period. Mortality data for all nine experimental treatments, including the two excluded treatments, are presented in Supplementary Table S7. In the soybean grits treatment, pronounced cannibalistic behavior was recorded, resulting in a drastic reduction in individual numbers and the survival of only a few large larvae. Under these conditions, reliable monitoring of growth and feed utilization parameters was not feasible, and both treatments were therefore omitted from all subsequent analyses. The highest weights were observed in the control group fed with wheat bran, whereas all other treatments showed lower values. Table 2 presents the values for final individual weight, specific growth rate (SGR), feed intake, mortality, feed conversion ratio (FCR), and efficiency of conversion of ingested food (ECI) calculated on a natural dry matter basis. Although FCR and ECI are closely related measures of feed conversion efficiency, both were included because FCR is a conventional indicator in animal production, whereas ECI is widely used in insect research. The highest average individual weight was recorded in the wheat bran treatment (604 mg). In contrast, the average weights observed in the triticale, wheat, and sunflower meal treatments were relatively similar, reaching approximately 50–60% of the weight achieved in the wheat bran group. The poorest performance was observed in *Zophobas* larvae fed with breadcrumbs, DDGS, and canola meal. Feed conversion ratio varied considerably, ranging from 2.55 in the wheat bran treatment to 17.50 in the breadcrumb treatment.

**Figure 1:**
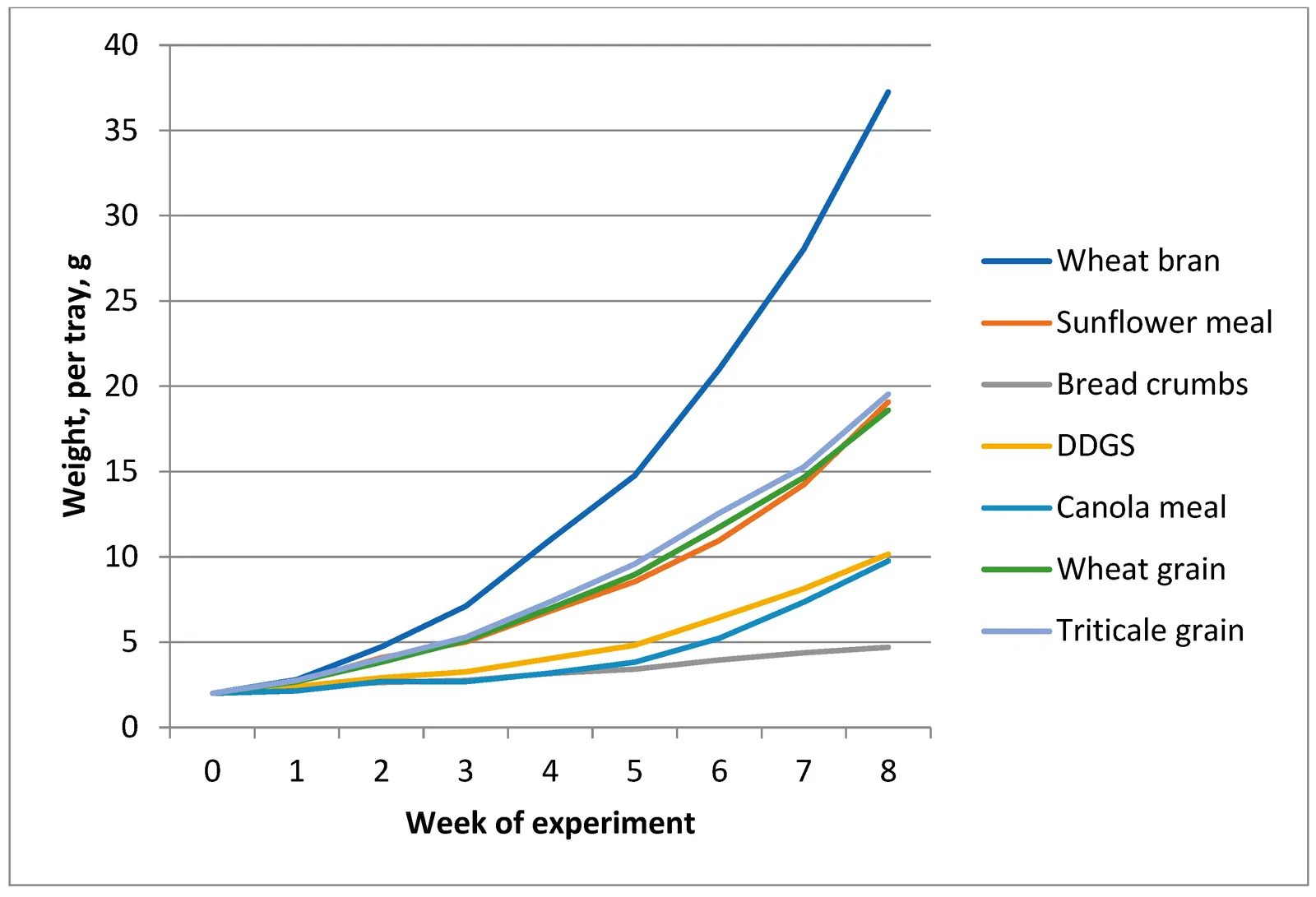
Effect of feedstuff type on mealworm (*Zophobas morio*) larval weight across experimental weeks. Data points represent treatment means for each time point.

**Figure 2:**
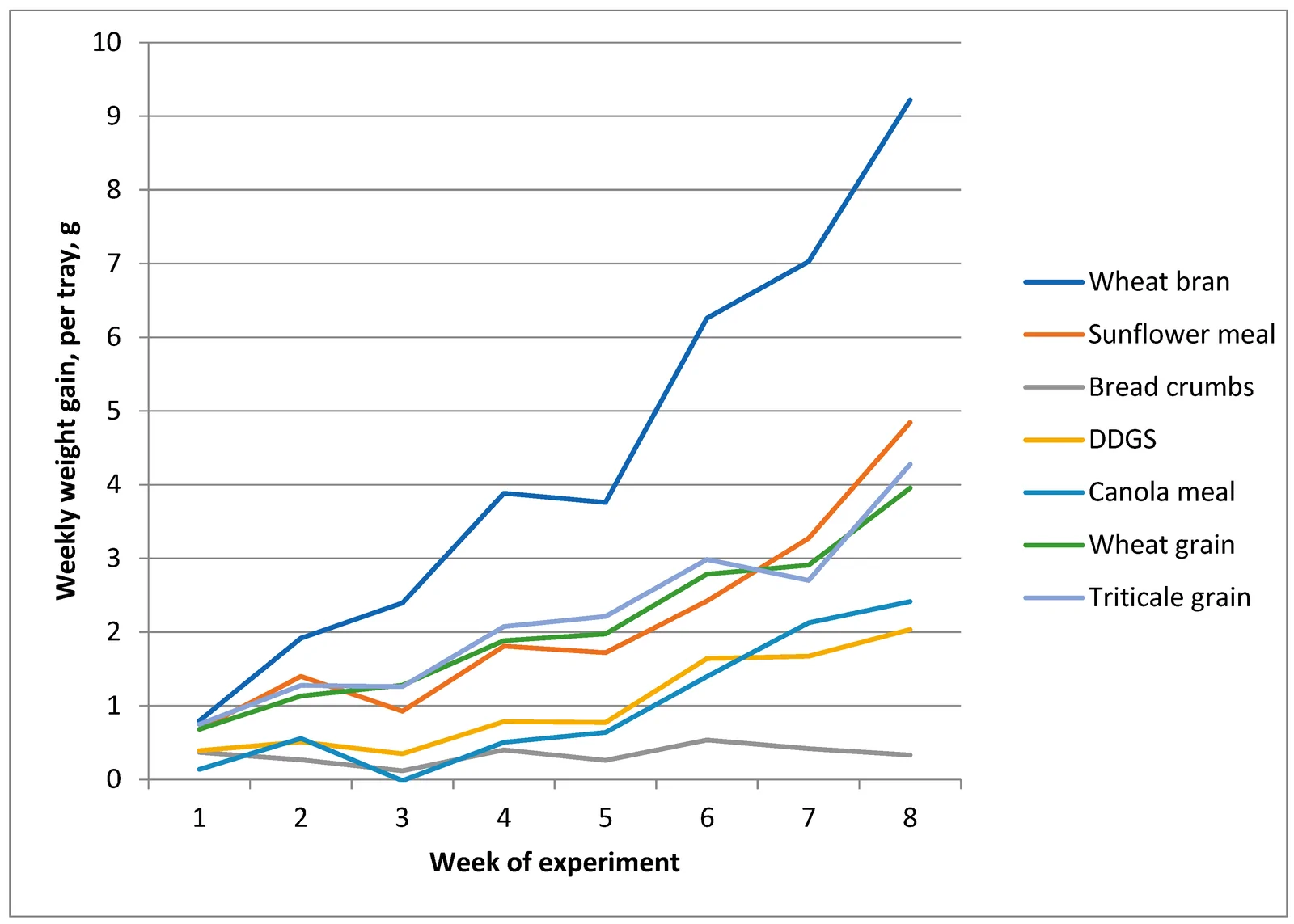
Effect of feedstuff type on mealworm (*Zophobas morio*) larval weight gain across experimental weeks. Data points represent treatment means for each time point.

**Figure 3:**
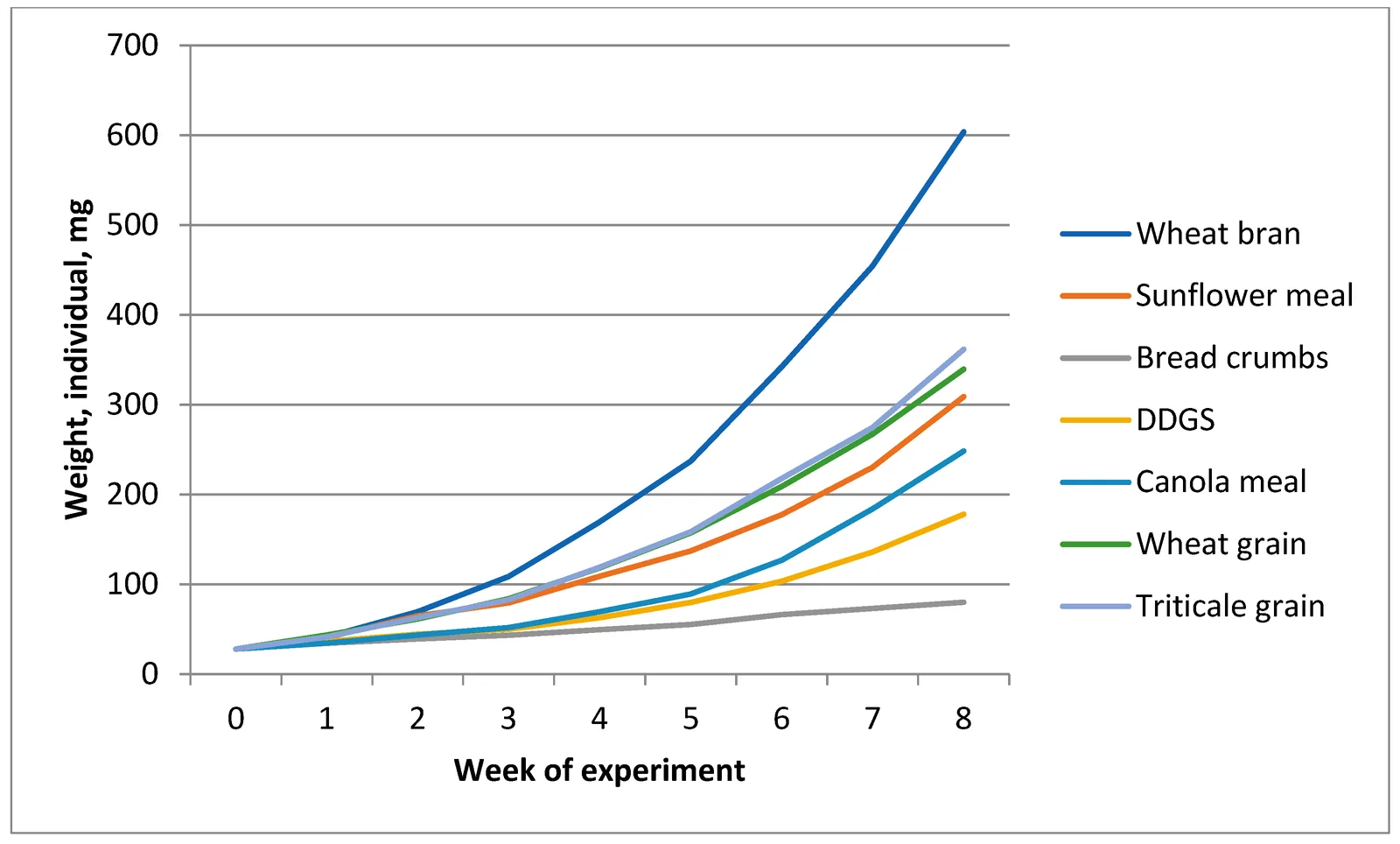
Effect of feedstuff type on individual superworm larvae (*Zophobas morio*) larval weight across experimental weeks. Data points represent treatment means for each time point.

**Figure 4:**
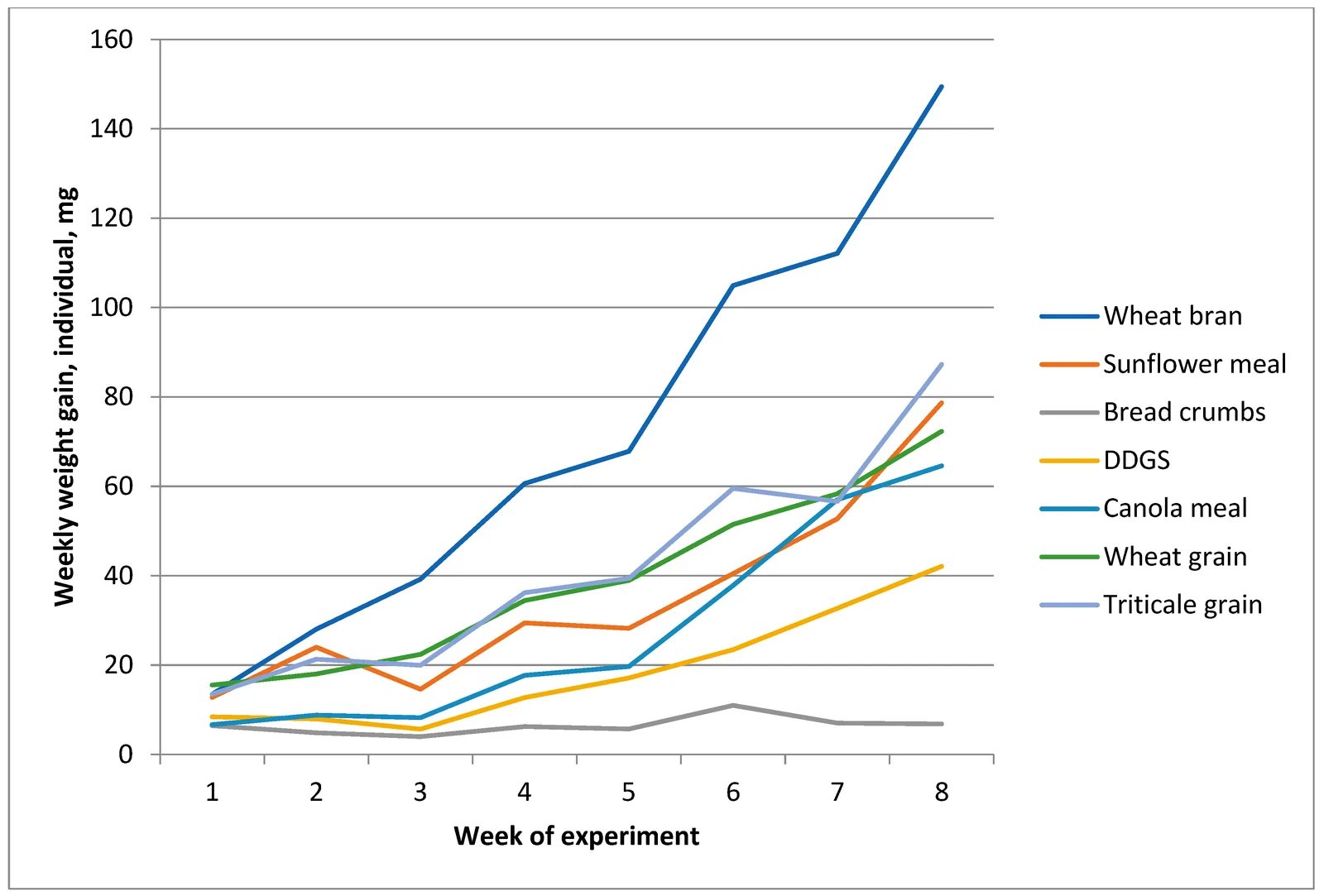
Effect of feedstuff type on individual superworm larvae (*Zophobas morio*) larval weight gain across experimental weeks. Data points represent treatment means for each time point.

**Figure 5:**
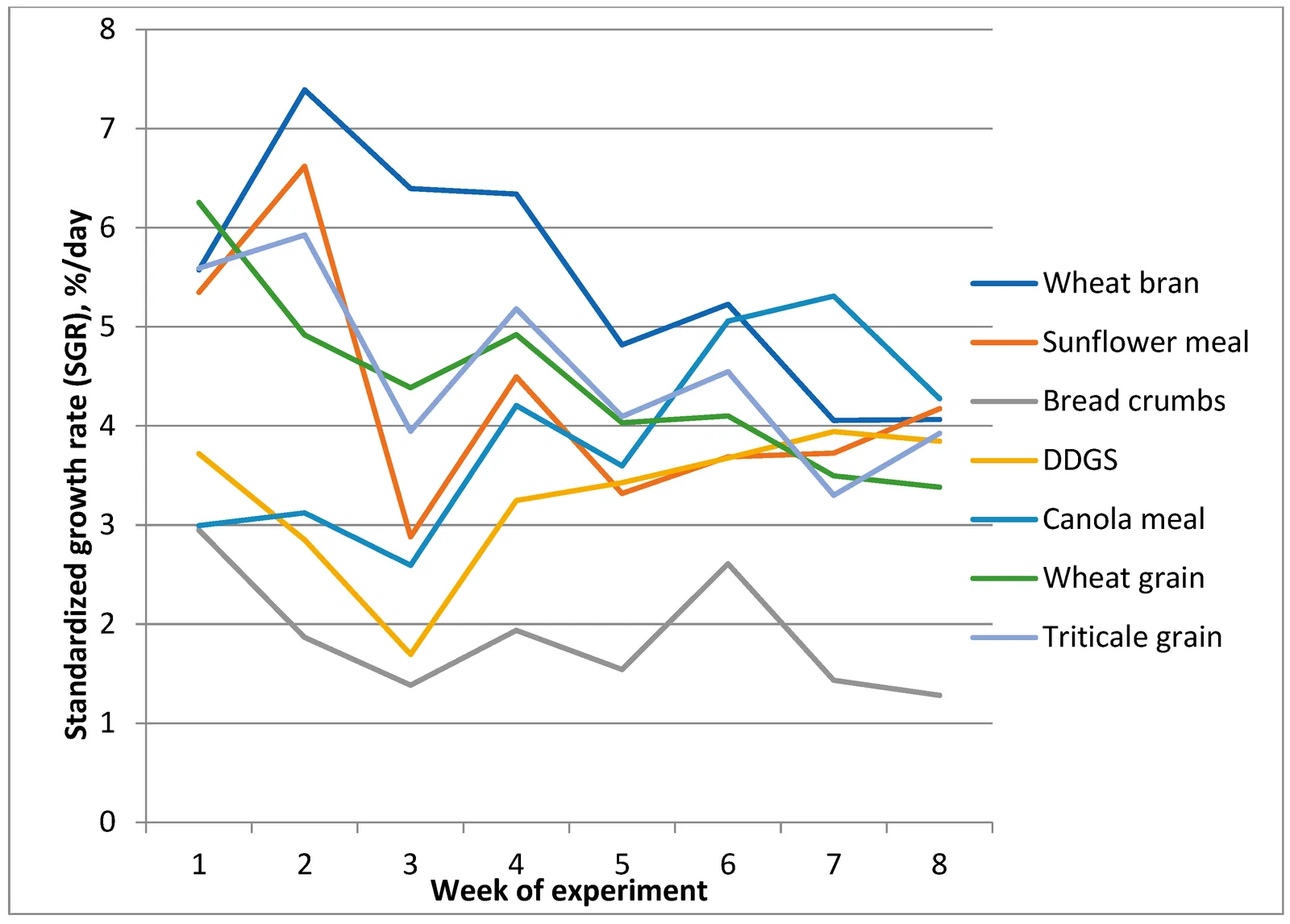
Effect of feedstuff type on superworm larvae (Zophobas morio) standardized growth rate (SGR) across experimental weeks. Data points represent treatment means for each time point.

**Figure 6:**
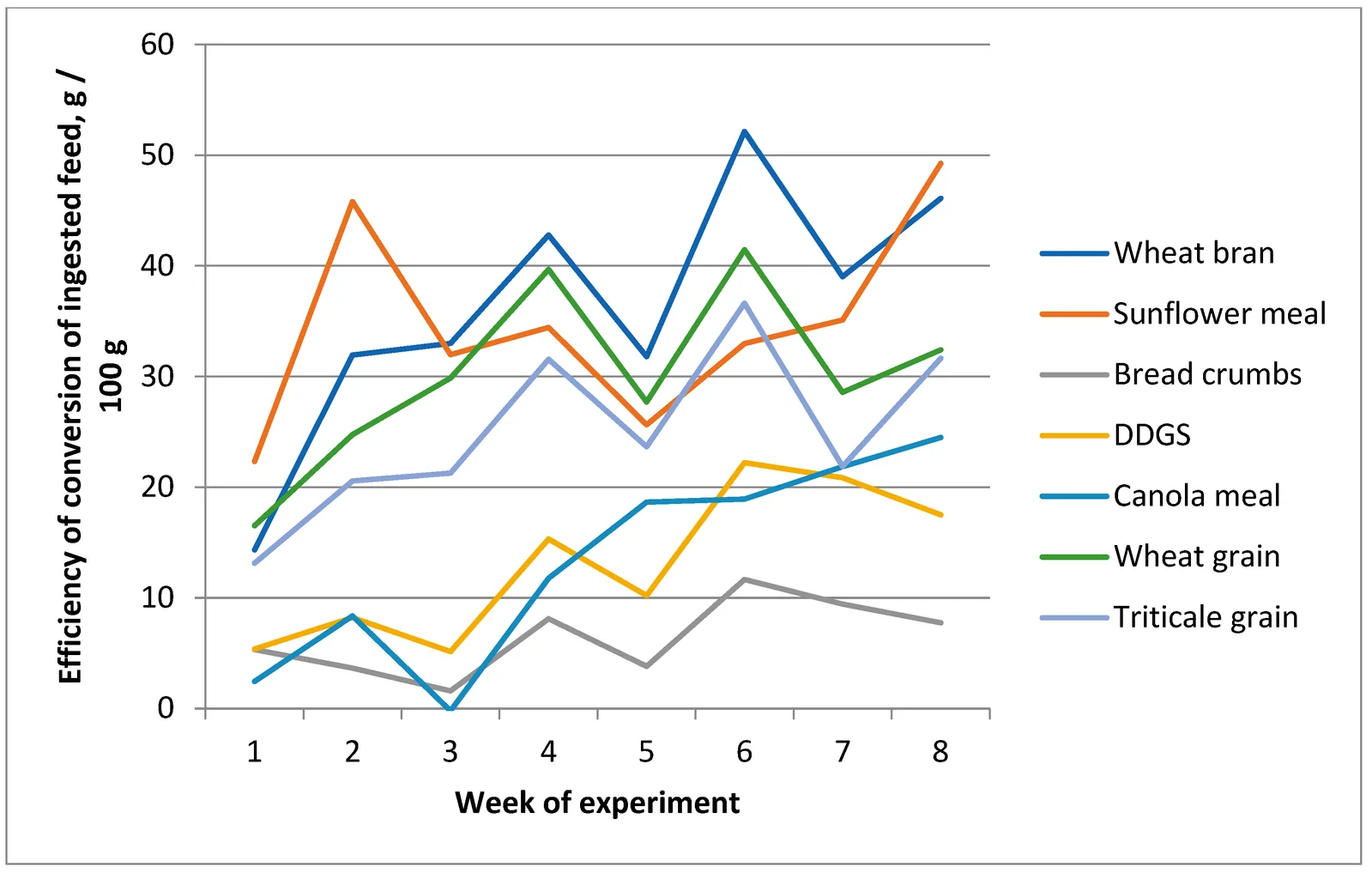
Effect of feedstuff type on superworm larvae (*Zophobas morio*) efficiency of conversion of ingested feed (ECI). Data points represent treatment means for each time point.

The results are presented in Figure 1, Figure 2, Figure 3, Figure 4, Figure 5 and Figure 6, illustrating the performance of different feeds. The corresponding weekly data, including means and standard deviations for all production performance parameters, are provided in Supplementary Table S1–S6. Wheat bran (Figure 1, Figure 2, Figure 3, Figure 4, Figure 5 and Figure 6) consistently showed the best overall performance in terms of both individual growth and growth per container and weight gain per tray and per individual larva (Figure 1, Figure 2, Figure 3 and Figure 4) although some feeds occasionally performed better at certain time points during the observation period. Similar trends were observed for specific growth rate (Figure 5) and efficiency of conversion of ingested feed (Figure 6), although some feeds occasionally performed better at certain time points during the observation period.

Although breadcrumbs are based on wheat flour, the larvae did not accept this feed. This rejection may have been associated with unfavorable physical characteristics, particularly excessive dryness and the presence of hard particles, which may have reduced palatability and hindered feed intake. Due to the negligible feed consumption, it was not possible to determine dry matter digestibility, digestible energy, or the energy digestibility coefficient for this feed. Therefore, the lack of measurable nutritional parameters can be considered a direct consequence of feed refusal, further emphasizing the importance of physical feed properties in determining feeding behavior and performance of larvae, beyond chemical composition alone.

## 4. Discussion

*Zophobas morio* appears to be a promising insect species for intensive production, particularly because its performance can be supported by a range of plant-derived feed materials. The suitability of ingredients such as wheat and triticale is of practical interest because these cereals are widely available and are primarily used as sources of carbohydrates in animal nutrition. More importantly, the response to sunflower meal provides an interesting perspective for diet formulation. Despite its relatively high fibre content, sunflower meal supported relatively good production performance, suggesting that a relatively high fibre content alone may not necessarily preclude the use of a plant-derived ingredient in *Z. morio* diets. This may be particularly relevant for the development of more diverse and economically feasible insect feeds, where ingredients with different nutritional characteristics can be combined rather than relying predominantly on conventional substrates such as wheat bran. Overall, the findings provide a basis for moving from the evaluation of individual feed ingredients towards the formulation of balanced feed mixtures specifically designed to meet the nutritional requirements of *Z. morio*. Such an approach could improve the flexibility of feed formulation and contribute to the development of more efficient and sustainable intensive production systems.

Of the nine diets evaluated, two proved unsuitable for the experimental rearing of *Zophobas morio* because they were associated with pronounced adverse responses, including the occurrence of cannibalistic behaviour and a sudden increase in mortality. Although the underlying causes of these responses cannot be conclusively determined based on the present results, such effects are of particular importance for intensive production systems, where reduced survival and behavioural disturbances could substantially compromise overall production efficiency. These observations therefore indicate that caution should be exercised when incorporating high proportions of certain feed ingredients into *Z. morio* diets. The observed response may be associated with nutritional imbalance, particularly a limited supply of readily utilizable energy relative to protein content, which could promote cannibalistic behaviour under stressful conditions, as previously suggested by [29].

In contrast, larvae fed field pea exhibited a different response from those fed soybean grits; however, a sudden and substantial increase in mortality was observed during the third week, leading to the termination of this treatment. As larvae were provided with an adequate water source (agar), dehydration can be excluded as a cause. The observed mortality may have been associated with the limited nutritional suitability of raw field pea as a sole diet, including reduced nutrient availability and the presence of antinutritional factors, such as trypsin inhibitors, potentially resulting in functional starvation.

The data obtained for soybean grits and field pea indicate that both nutritional imbalance and the presence of antinutritional compounds can severely impair the survival and production performance of *Zophobas morio* larvae, emphasizing the importance of balanced diets and appropriate feed processing in intensive rearing systems. High-protein feed substrates may influence production performance parameters in tenebrionid insects [30].

Comparison with previously published data is extremely difficult due to the limited number of available studies and the frequent use of different feed substrates. In the study by Gourgouta et al. 2024 [17], two feedstuffs identical to those used in the present experiment, wheat bran and sunflower meal, were evaluated. Our results (Table 2) showed that the feed conversion ratio (FCR) was 2.55 for wheat bran and 2.80 for sunflower meal, which is very similar to the values reported by Gourgouta et al. 2024 [17], where FCR amounted to 2.4 and 3.1, respectively. The largest number of available studies concerns wheat bran, as it is commonly used as a control diet in insect feeding experiments. The results obtained in the present study for wheat bran were within the range reported in the literature [18,24]. However, comparison of the remaining feedstuffs with published data is considerably more difficult due to the lack of comparable studies. Interestingly, feed conversion ratios for wheat and triticale were considerably higher, reaching 3.28 and 3.87, respectively. The lowest mortality was observed in larvae fed sunflower meal. The relatively good performance observed with fibre-rich feedstuffs suggests that feed materials traditionally considered less suitable for insect production should not necessarily be excluded from consideration. Instead, such materials may have potential when combined with more conventional substrates, such as wheat bran, allowing the development of diets that combine different nutritional characteristics while maintaining adequate larval growth.

It should be emphasized that the present results, expressed on both a per-tray and individual larval weight basis, reflect the performance of *Zophobas morio* when individual feedstuffs were used as sole dietary substrates. Such substrates may not provide a nutritionally balanced supply of all essential nutrients; therefore, poorer performance should not be interpreted as an indication that these feedstuffs are unsuitable for inclusion in nutritionally balanced formulated diets. These findings may broaden the range of feed materials considered for *Z. morio* production, particularly by encouraging the evaluation of fibre-rich and unconventional plant-derived substrates that might otherwise be overlooked.

To the best of our knowledge, data on the digestibility and the energy digestibility coefficient (i.e., the proportion of ingested gross energy remaining after fecal losses) of *Zophobas morio* are currently lacking. Digestibility trials in insects present particular methodological challenges, as larvae are continuously exposed to and interact with both their feed and frass, making complete separation of excreta from residual feed difficult. Nevertheless, further development of digestibility methodologies and accumulation of such data are important for improving our understanding of nutrient utilization and supporting the optimization of intensive insect production systems. The results of the present study are presented in Table 3. It is interesting that in the study by Pascual et al. (2024) [21], which investigated apparent digestibility in *Tenebrio molitor*, only one feedstuff overlaps with our experiment—wheat bran. It should be noted that digestibility coefficients in the present study are expressed on a decimal scale (0–1), whereas Pascual et al., 2024 [21] reported the same parameters as “digestibility coefficients (%)”, i.e., in percentage form. Dry matter digestibility of wheat bran was very similar between studies, with 0.407 (40.7%) reported by Pascual et al. and 0.43 obtained in our experiment. The digestible energy values were also comparable, with 8.66 MJ/kg DM reported [21] and 8.50 MJ/kg DM obtained in our study, indicating consistent energy utilization of this feedstuff across experiments. Regarding the other feedstuffs, the highest the digestible energy in our study was recorded for wheat (10.40 MJ/kg DM), which is expected due to its high starch content as an energy source. In contrast, the lowest value was observed for canola meal (5.54 MJ/kg DM), which also showed the poor digestibility. Although larvae showed good growth on sunflower-based diets, digestibility of dry matter was relatively low (0.31), which may be attributed to the high fibre content and structural carbohydrates (including cellulose and lignin) present in sunflower hulls.

There are limited data regarding the influence of diet on the chemical composition of *Zophobas morio* larvae. The results of the chemical composition of larvae depending on diet are presented in Table 4. In insect species such as *Tenebrio molitor*, a clear relationship between diet and chemical composition has been established, with the protein and lipid contents of larvae showing considerable variation depending on the feed substrate [31,32,33,34,35]. One of the challenges in insect rearing is that dietary differences may affect the rate of development, resulting in larvae from different dietary treatments reaching different physiological stages at the same chronological age, which may in turn affect their body composition. However, available data on *Zophobas morio* remain scarce. In study [17] different feed materials and feed mixtures had a pronounced effect on the chemical composition of the larvae. For certain feedstuffs, such as sunflower meal, which was also used in our experiment, the obtained results were very similar to ours. However, when larvae were fed mixtures that were isonitrogenic, numerical differences in larval protein content were observed. In our study, in which single feed ingredients were used, the chemical composition of the larvae varied significantly depending on the diet. The highest protein content, expressed on a dry matter basis, was recorded in the group fed sunflower meal, whereas the lowest protein content was observed in larvae fed breadcrumbs. Our results regarding the protein content of larvae fed sunflower meal are consistent with those reported by Gourgouta et al. 2024 [17]. Similar agreement was observed for lipid content, with 27.45% lipids recorded in our study compared to 27.8% reported in theirs. Since several of the feeds used in our experiment were rich in starch, an important energy source (triticale, wheat, and breadcrumbs), larvae fed these diets were associated with the highest lipid contents. Nevertheless, relatively high lipid levels were also observed in larvae fed DDGS, despite this feedstuff being characterized by a high protein content. In our study, NDF and ADF fractions were determined. The ADF fraction was affected by diet, with the highest values observed in larvae fed wheat-based diets. This is of particular relevance, as the ADF fraction may be relevant to the nutritional quality of the final insect product, as higher ADF levels have been associated with reduced in vitro protein digestibility of insect meals [35].

## 5. Conclusions

This study demonstrates that the production performance of *Zophobas morio* is strongly dependent on the feed substrate, as assessed both through individual larval weight and under production-relevant conditions. Nutritional, physical, and chemical properties of the feed have contributed to differences in growth, digestibility, and larval composition. In addition, certain substrates induced behavioral changes, such as reduced feed intake or cannibalism, which further affected production outcomes. Overall, the results indicate that feed choice can affect the production performance of *Zophobas morio*, with some less commonly used feed ingredients, such as sunflower meal, showing promising potential. These findings suggest that fibre-rich and other less commonly used plant-derived feed materials may have potential as components of *Zophobas morio* diets, thereby broadening the range of alternative feed materials that could be considered for insect production.

## Figures and Tables

**Table 1:** Composition of the feedstuffs in their natural dry state used in the trial with *Zophobas morio*.

	Dry Matter	Crude Protein	Ether Extract	CF	Ash	NFE	Gross Energy (MJ/kg)
**Wheat bran**	90.19	18.04	4.00	8.28	4.80	55.07	18.14
**Soybean grits**	93.81	38.73	20.45	4.81	5.43	24.39	18.06
**DDGS**	89.88	32.25	4.68	6.93	4.86	41.16	20.07
**Field peas**	89.63	22.27	1.41	4.93	3.00	58.02	18.70
**Canola meal**	90.48	32.54	8.59	11.70	5.22	32.43	20.34
**Sunflower meal**	90.71	39.7	1.14	18.37	6.41	25.12	18.06
**breadcrumbs**	90.85	11.27	2.23	0.32	2.76	74.27	18.55
**wheat**	89.6	13.82	1.53	2.31	1.39	70.55	17.19
**triticale**	90.41	11.37	1.63	2.82	1.89	72.7	17.18

CF—crude fibre; NFE—nitrogen-free extract.

**Table 2:** Final individual weight, standardized growth rate (SGR), feed intake, mortality, feed conversion ratio (FCR) and efficiency of conversion of ingested feed (ECI) of superworm larvae (*Zophobas morio*) fed seven different feedstuffs (mean ± SD).

	Final Individual Weight, mg	SGR, %/Day	Feed Intake, per Tray, g	Mortality, %	FCR, g/g	ECI, %
**Wheat bran**	604 ± 13 ^a^	5.48 ± 0.04 ^a^	89.7 ± 0.6 ^a^	9.7 ± 5.2 ^a^	2.55 ± 0.09 ^a^	39.3 ± 1.3 ^a^
**Sunflower meal**	309 ± 29 ^bc^	4.28 ± 0.17 ^b^	47.6 ± 2.9 ^e^	8.2 ± 2.9 ^a^	2.80 ± 0.20 ^ab^	35.9 ± 2.5 ^a^
**Breadcrumbs**	80 ± 3 ^e^	1.88 ± 0.06 ^e^	47.2 ± 1.5 ^e^	15.1 ± 4.3 ^a^	17.50 ± 0.94 ^d^	5.7 ± 0.3 ^e^
**DDGS**	178 ± 13 ^d^	3.30 ± 0.12 ^d^	60.4 ± 1.2 ^c^	15.3 ± 5.3 ^a^	7.45 ± 0.65 ^c^	13.5 ± 1.3 ^d^
**Canola meal**	249 ± 20 ^c^	3.89 ± 0.14 ^c^	55.8 ± 2.0 ^d^	41.4 ± 8.6 ^b^	7.22 ± 0.57 ^c^	13.9 ± 1.1 ^d^
**Wheat grain**	340 ± 57 ^b^	4.44 ± 0.30 ^b^	54.1 ± 1.0 ^d^	14.6 ± 11.9 ^a^	3.28 ± 0.29 ^ab^	30.7 ± 2.6 ^b^
**Triticale grain**	362 ± 30 ^b^	4.56 ± 0.15 ^b^	67.8 ± 1.1 ^b^	20.5 ± 4.2 ^a^	3.87 ± 0.12 ^b^	22.8 ± 1.0 ^c^

Values are presented as mean ± SD (*n* = 4); ^a,b,c,d,e^ Means that do not share a common letter are considered significantly different (*p* < 0.05).

**Table 3:** The apparent tract digestibility coefficient of dry matter, digestible energy, and apparent energy digestibility coefficient of different feedstuffs fed to superworm larvae (*Zophobas morio*) (mean ± SD).

	Dry Matter Digestibility	Digestible Energy, MJ/kg Dry Matter	Apparent Energy Digestibility Coefficient
Wheat bran	0.43 ± 0.02 ^b^	8.50 ± 0.52 ^c^	0.45 ± 0.03 ^b^
Sunflower meal	0.31 ± 0.03 ^c^	8.58 ± 0.50 ^bc^	0.43 ± 0.03 ^b^
Breadcrumbs	- *	-	-
DDGS	0.30 ± 0.01 ^c^	8.41 ± 0.41 ^c^	0.38 ± 0.02 ^c^
Canola meal	0.16 ± 0.03 ^d^	5.54 ± 0.49 ^d^	0.25 ± 0.02 ^d^
Wheat grain	0.52 ± 0.01 ^a^	10.40 ± 0.14 ^a^	0.54 ± 0.01 ^a^
Triticale grain	0.49 ± 0.03 ^a^	9.62 ± 0.59 ^ab^	0.51 ± 0.03 ^a^

^a,b,c,d^ Means that do not share a common letter are considered significantly different (*p* < 0.05), * Feed intake in breadcrumbs fed larvae was too low to allow for excreta collection needed for digestibility trial.

**Table 4:** Effect of diet on the chemical composition of *Zophobas morio* (% of dry matter) (mean ± SD).

	Crude Protein	Ether Extract	Ash	Crude Fiber	ADF	NDF	ADL
**Wheat bran**	46.69 ± 0.02 ^e^	41.40 ± 0.59 ^d^	2.66 ± 0.01 ^d^	5.18 ± 0.03 ^a^	5.34 ± 0.06 ^a^	8.27 ± 0.49 ^ab^	0.48 ± 0.06 ^b^
**Sunflower meal**	59.79 ± 0.24 ^g^	27.45 ± 0.41 ^b^	2.97 ± 0.04 ^b^	5.67 ± 0.05 ^cd^	7.46 ± 0.46 ^d^	7.86 ± 0.15 ^b^	0.66 ± 0.01 ^ab^
**Breadcrumbs**	28.85 ± 0.15 ^a^	60.44 ± 0.29 ^f^	2.23 ± 0.03 ^c^	4.53 ± 0.11 ^b^	4.69 ± 0.04 ^a^	6.24 ± 0.05 ^c^	0.67 ± 0.05 ^ab^
**DDGS**	41.23 ± 0.31 ^d^	46.39 ± 0.71 ^e^	2.55 ± 0.09 ^a^	5.10 ± 0.17 ^a^	7.33 ± 0.23 ^cd^	6.20 ± 0.15 ^ab^	0.77 ± 0.07 ^a^
**Canola meal**	52.40 ± 0.25 ^f^	37.97 ± 0.54 ^c^	2.85 ± 0.026 ^b^	5.32 ± 0.19 ^ac^	6.28 ± 0.34 ^bc^	8.50 ± 0.33 ^ab^	0.76 ± 0.06 ^a^
**Wheat grain**	37.31 ± 0.05 ^c^	53.65 ± 1.04 ^a^	2.49 ± 0.02 ^a^	6.03 ± 0.32 ^d^	9.89 ± 0.015 ^f^	7.89 ± 0.38 ^a^	0.58 ± 0.02 ^ab^
**Triticale grain**	34.92 ± 0.26 ^b^	55.78 ± 0.89 ^a^	2.46 ± 0.02 ^a^	5.04 ± 0.17 ^ab^	6.29 ± 0.23 ^b^	8.29 ± 0.12 ^ab^	0.70 ± 0.11 ^a^

^a,b,c,d,e,f,g^ Means that do not share a common letter are considered significantly different (*p* < 0.05).

## Data Availability

The original contributions presented in this study are included in the article. Further inquiries can be directed to the corresponding author.

## Supplementary Materials

The following supporting information can be downloaded at: https://www.mdpi.com/article/10.3390/insects17101049/s1, Table S1–S6: Weekly production performance parameters of *Zophobas morio* larvae fed seven different feedstuffs; Table S7: Larval mortality across nine experimental treatments.
