# Supplementary material for: The Effects of Different Feed Substrates on Production Performance, Digestibility, and Chemical Composition of Zophobas morio Larvae
Source: Insects. 2026 Oct 12;17(10):1049. doi: 10.3390/insects17101049 (PMC13705041; doi:10.3390/insects17101049)
Supplement: Supplementary file 1 [file insects-17-01049-s001.zip › insects-4499080-supplementary.pdf]

Tables for Supplementary Materials

Table S1. Weekly larval weights per tray (g) of mealworm larvae (*Zophobas morio*) fed seven differen feedstuffs (Mean ± SD)

	Wheat bran	Sunflower meal	Bread crumbs	DDGS	Canola meal	Wheat grain	Triticale grain
Week 1	2.80±0.04	2.68±0.02	2.37±0.05	2.39±0.04	2.14±0.13	2.68±0.04	2.75±0.03
Week 2	4.72±0.19	4.08±0.18	2.64±0.08	2.90±0.04	2.70±0.35	3.82±0.17	4.03±0.11
Week 3	7.11±0.35	5.01±0.43	2.76±0.12	3.25±0.03	2.68±0.27	5.10±0.23	5.29±0.13
Week 4	11.00±0.41	6.82±0.33	3.16±0.08	4.04±0.14	3.19±0.30	6.98±0.21	7.36±0.28
Week 5	14.76±0.43	8.54±0.35	3.42±0.11	4.81±0.13	3.82±0.18	8.95±0.14	9.58±0.37
Week 6	21.01±0.50	10.96±0.44	3.95±0.09	6.45±0.12	5.22±0.20	11.74±0.37	12.56±0.59
Week 7	28.04±1.03	14.23±0.62	4.37±0.18	8.12±0.41	7.35±0.38	14.65±0.35	15.26±0.70
Week 8	37.26±1.30	19.08±1.25	4.70±0.12	10.16±0.70	9.76±0.47	18.60±1.51	19.54±0.66

Table S2. Weekly larval weight gains per tray (g/week) of mealworm larvae (*Zophobas morio*) fed seven differen feedstuffs (Mean ± SD)

	Wheat bran	Sunflower meal	Bread crumbs	DDGS	Canola meal	Wheat grain	Triticale grain
Week 1	0.80±0.04	0.68±0.02	0.37±0.05	0.39±0.03	0.14±0.13	0.68±0.04	0.75±0.03
Week 2	1.92±0.20	1.40±0.18	0.27±0.07	0.51±0.04	0.56±0.22	1.13±0.15	1.28±0.08
Week 3	2.40±0.17	0.93±0.32	0.12±0.15	0.35±0.05	-0.01±0.11	1.28±0.19	1.26±0.07
Week 4	3.89±0.31	1.81±0.14	0.40±0.08	0.79±0.12	0.51±0.17	1.88±0.13	2.08±0.24
Week 5	3.76±0.35	1.72±0.19	0.26±0.03	0.77±0.03	0.64±0.13	1.98±0.07	2.21±0.16
Week 6	6.26±0.27	2.42±0.13	0.54±0.04	1.64±0.07	1.40±0.14	2.79±0.43	2.99±0.41
Week 7	7.03±0.73	3.27±0.27	0.42±0.11	1.67±0.35	2.13±0.20	2.91±0.11	2.70±0.47
Week 8	9.22±0.52	4.85±0.91	0.33±0.09	2.04±0.55	2.42±0.17	3.96±1.30	4.28±0.56

Table S3. Weekly individual larval weights (mg) of mealworm larvae (*Zophobas morio*) fed seven differen feedstuffs (Mean ± SD)

	Wheat bran	Sunflower meal	Bread crumbs	DDGS	Canola meal	Wheat grain	Triticale grain
Week 1	41±3	41±3	34±0	36±3	35±4	44±4	41±3
Week 2	70±4	65±4	39±2	44±2	44±8	62±7	63±3
Week 3	109±5	79±6	43±2	50±4	52±5	84±13	83±4
Week 4	169±6	109±10	50±2	63±3	69±7	118±17	119±6
Week 5	237±5	137±8	55±3	80±6	89±6	157±26	158±7
Week 6	342±14	178±13	66±3	103±8	127±7	209±27	218±13
Week 7	454±11	230±13	73±3	136±8	184±7	267±39	275±16
Week 8	604±13	309±29	80±3	178±13	249±20	340±57	362±30

Table S4. Weekly individual larval weight gains (mg/week) of mealworm larvae (*Zophobas morio*) fed seven differen feedstuffs (Mean ± SD)

	Wheat bran	Sunflower meal	Bread crumbs	DDGS	Canola meal	Wheat grain	Triticale grain
Week 1	13.5±3.5	12.8±2.9	6.4±0.3	8.4±3.1	6.7±3.8	15.5±4.1	13.5±2.8
Week 2	28.0±1.9	24.0±1.0	4.8±2.0	8.0±3.4	8.8±8.0	18.0±7.6	21.3±2.8
Week 3	39.2±3.4	14.6±5.0	4.0±1.2	5.7±3.2	8.2±6.0	22.4±7.0	20.0±1.6
Week 4	60.6±1.9	29.4±5.3	6.3±1.0	12.7±1.0	17.7±5.5	34.4±4.9	36.2±2.6
Week 5	67.8±4.7	28.2±2.3	5.7±1.7	17.1±5.3	19.7±5.9	39.0±8.9	39.4±4.8
Week 6	105.0±10.6	40.5±4.8	11.0±1.7	23.4±8.6	37.8±2.9	51.5±2.5	59.5±6.9
Week 7	112.1±7.8	52.7±2.0	7.0±2.2	32.7±2.9	57.0±1.7	58.3±13.5	56.6±9.9
Week 8	149.5±8.4	78.7±17.5	6.8±2.9	42.1±5.1	64.6±14.3	72.3±24.5	87.3±14.5

Table S5. Weekly standardized growth rate (SGR, %/day) of mealworm larvae (*Zophobas morio*) fed seven differen feedstuffs (Mean ± SD)

	Wheat bran	Sunflower meal	Bread crumbs	DDGS	Canola meal	Wheat grain	Triticale grain
Week 1	5.57±1.17	5.35±1.05	2.95±0.11	3.72±1.22	2.99±1.59	6.26±1.29	5.59±0.96
Week 2	7.39±0.62	6.62±0.32	1.87±0.75	2.85±1.32	3.12±2.61	4.92±1.89	5.93±0.80
Week 3	6.39±0.52	2.88±0.90	1.38±0.38	1.69±0.93	2.59±1.85	4.38±0.92	3.95±0.34
Week 4	6.34±0.28	4.50±0.66	1.94±0.38	3.25±0.38	4.21±1.20	4.92±0.27	5.18±0.12
Week 5	4.82±0.38	3.32±0.46	1.54±0.38	3.43±0.98	3.60±1.12	4.03±0.29	4.09±0.52
Week 6	5.23±0.39	3.69±0.23	2.61±0.48	3.68±1.31	5.06±0.35	4.10±0.53	4.55±0.32
Week 7	4.06±0.37	3.72±0.22	1.43±0.45	3.94±0.45	5.31±0.30	3.50±0.41	3.30±0.55
Week 8	4.06±0.23	4.17±0.67	1.28±0.57	3.85±0.31	4.27±0.68	3.38±0.98	3.92±0.42

Table S6. Weekly efficiency of conversion of ingested feed (ECI, g/100g) of mealworm larvae (*Zophobas morio*) fed seven differen feedstuffs (Mean ± SD)

	Wheat bran	Sunflower meal	Bread crumbs	DDGS	Canola meal	Wheat grain	Triticale grain
Week 1	14.34±0.88	22.33±2.64	5.32±0.45	5.38±1.09	2.44±2.34	16.52±1.78	13.14±1.29
Week 2	31.93±2.74	45.81±3.03	3.65±0.99	8.22±0.81	8.36±3.39	24.74±6.42	20.57±1.46
Week 3	33.00±1.24	31.97±9.38	1.59±2.04	5.15±0.73	-0.21±1.61	29.86±4.60	21.26±1.07
Week 4	42.79±2.65	34.44±5.08	8.11±2.97	15.32±4.54	11.77±5.18	39.68±1.37	31.55±3.23
Week 5	31.78±2.76	25.63±1.78	3.80±0.61	10.19±0.88	18.65±13.63	27.70±1.00	23.66±1.54
Week 6	52.15±2.22	32.97±1.00	11.66±1.66	22.22±0.58	18.91±1.36	41.47±5.19	36.63±5.74
Week 7	39.03±4.08	35.11±2.61	9.44±2.61	20.84±4.51	21.84±1.95	28.57±1.63	21.87±3.34
Week 8	46.10±2.59	49.27±11.75	7.75±2.20	17.51±4.49	24.50±1.83	32.42±9.90	31.65±4.65

Table S7. Mortality of mealworm larvae (*Zophobas morio*) fed seven differen feedstuffs included in final analysis and two feedstuffs that were excluded (Mean ± SD)

	Mortality, %
Wheat bran	9.7 ± 5.2
Sunflower meal	8.2 ± 2.9
Bread crumbs	15.1 ± 4.3
DDGS	15.3 ± 5.3
Canola meal	41.4 ± 8.6
Wheat grain	14.6 ± 11.9
Triticale grain	20.5 ± 4.2
Soybean grits	75.1 ± 12.0
Field pea	34.4 ± 10.1
